# Effect of Laser Parameters on Fracture Properties of Laser-Repaired Cracks with Micro/NanoMaterial Addition: Multiscale Analysis

**DOI:** 10.3390/ma17184656

**Published:** 2024-09-23

**Authors:** Yinyin Li, Wei Jiang, Meiqiu Li

**Affiliations:** 1 School of Mechanical Engineering, Yangtze University, Jingzhou 434023, China; liyyhbsy@yangtzeu.edu.cn (Y.L.); limq@yangtzeu.edu.cn (M.L.); 2 School of Mechanical Engineering, Dalian University of Technology, Dalian 116024, China

**Keywords:** laser process parameter, microstructure, fracture properties, crystal plasticity, cumulative plastic slip

## Abstract

In laser crack repair processes, laser parameters have significant influence on repair quality. Improper combination of laser process parameters may result in defects—such as porosity, ablation, and coarse grain size—in remelted zones. A trans-scale computational model is established by combining crystal plasticity finite elements and variable-node finite elements. The influence of microstructure characteristics such as grain size and porosity of the repair layer on the cumulative plastic slip (CPS) on the dominant slip system at the meso-scale and the J-integral at the macro-scale is studied to explore the effect of laser process parameters on repair quality. The results show that when the laser power is 1800 W and the heating time is 0.5 s, the grain size and porosity of the repaired specimen are the smallest. The J-integral of the repaired specimen is more than 8% smaller than that of the unrepaired specimen and about 3% smaller than that of the repaired specimen, with a laser power of 2000 W and a heating time of 1 s. Pores increase the CPS of the crystal around the pores, especially when a pore have sharp corners. Selecting appropriate laser process parameters can not only refine grain size but also reduce the volume fraction of pores and thus reduce the J-integral and eventually improve repair quality of repaired specimens. The study investigates the relationship of process parameter–microstructure–repair quality in the laser repair process and provides a method for studying the mechanical behavior of materials at macro and micro scales.

## 1. Introduction

As one of common failure modes in engineering structures, fractures widely exist in aerospace, automobile, ship, and petroleum engineering. The presence of cracks not only reduces the bearing capacity of materials, but may even cause catastrophic disasters, resulting in huge losses of life and economy. Therefore, early-stage crack repair could provide substantial savings for both resources and economy [1].

Laser repair is a feasible method of repairing cracks via metallurgical combination of suitable materials and the workpiece to be repaired with high-energy laser beam [2]. Wang et al. [3] studied the microstructure and wear resistance of titanium alloy components repaired with laser cladding and found that repair coatings show similar microhardness and wear resistance compared with the substrate. Cha et al. [4] proposed a novel system to repair aeroengine airfoil by pulsed laser ablation to remove surface cracks in damaged zone. After repairing, an equivalent performance of high cycle fatigue strength and less microstructural damage can be achieved compared to traditional mechanical material removal methods.

The selection of laser process parameters is a key issue in laser repair of cracks. Laser repair is a dynamic physical metallurgical process involving the interaction between laser, powder, and substrate. During the interaction between laser and materials, the temperature and flow fields in the molten pool affect the convection, solidification process, composition distribution, and microstructure, thus affecting the quality of the laser-repaired layer. Therefore, it is of great significance to choose the appropriate laser process parameters to improve repair quality. The existence of pores is one of the factors that are very sensitive to laser process parameters in the repaired layer. When the packing density of added powders is low, the gas between the powders may dissolve in the molten pool [5]. Under the action of laser, the powder will melt and solidify rapidly, and the gas has no chance to escape from the molten pool. Pores are thus formed in the repaired layer. When excessive input energy is provided, gas bubbles will be generated due to vaporization of low-melting-point ingredients within the alloy. If gas bubbles do not have enough time to rise and escape from the molten pool, regular spherical pores will form. When the input energy is insufficient, the metal powder cannot be melted completely, resulting in irregular pores [6]. Khairallah et al. [7] revealed the formation mechanisms of pore defects in strong dynamic melt flow in laser powder bed fusion by a powder-scale model. Han et al. [8] studied the influence the porosity distribution and mechanical response in laser and metal inert gas hybrid welding of 6082-T6 aluminum alloy. It is found that when the porosity is 3%, the fracture feature of material changes from pure ductile to the mixed mode of ductile and brittle. Cracks initiate on the weld surface when joints have no pores and initiate near pores when pores exist in the joint. Pekok et al. [9] investigated the formation mechanisms and characteristics of porosity in aluminum 2024 alloy through selective laser melting using high and low energy densities and found that low energy density reduces grain size and increases the microhardness of alloys.

The microstructure of the repair layer near the crack tip has undergone significant changes after laser irradiation, which will inevitably affect the mechanical properties of the material. The crystal plastic finite element (CPFE) method has outstanding advantages in solving meso-scale mechanics problems, such as grain size effect, grain interaction, strain gradient effect, crystal high-temperature deformation, texture evolution, recrystallization, etc. [10,11,12,13,14,15]. The influence of microstructure on the fracture performance of the repaired parts is studied using the CPFE method on the micro scale, which is helpful to improve the laser repair quality. Wang et al. [16] investigated the coupling effects of columnar grains and residual stress on the mechanical behavior of 316L stainless steel formed by selective laser melting through CPFE methods. The simulation results can predict the experimental trends well. Zhang et al. [17] investigated the high-cycle and very-high-cycle fatigue properties of AlSi10Mg alloy prepared via selective laser melting using CFEM method and successfully analyzed the effects of pores and inclusions on the fatigue performance of the material. In the aspect of fracture mechanics, Kirane and Ghosh [18] studied the early crack initiation by a CPFE model. It was found that the load shedding between grains is the main reason for early crack initiation. He et al. [19] employed the CPFE method to predict the slip line distribution as well as the crack initiation location and propagation direction, with preference. The significant stress gradient at the crack tip highlights the crucial role of the microstructure in the vicinity of the crack tip in determining the fracture resistance of the repaired layer. By employing the CPFE method to investigate the influence of the microstructure near the crack tip on the fracture properties of the repaired layer, we can gain valuable insights into the laser repair mechanism of cracks.

Jiang et al. [20] studied the effect of laser process parameters on the repair effect of CT specimens with cracks through experiments; while Li et al. [21,22] established a macro–meso trans-scale model to study the macroscopic and microscopic mechanical behavior of materials. On the basis of these studies, this article analyzes the influence of laser process parameters on the microstructure of materials, with a focus on the influence of laser process parameters on porosity in the cladding layer. Then, a macro–meso trans-scale model is used to study the influence of microstructure such as grain size, pore distribution, and pore shape on the macro- and micromechanical properties of repaired parts. The results offer a theoretical guidance for selecting appropriate process parameters for laser repair of stainless steel materials with cracks.

## 2. Experiment and Models

### 2.1. Experiment and Materials

**Figure 1:**
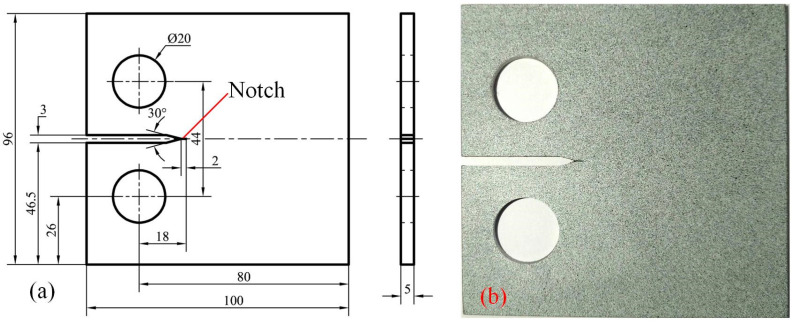
(**a**) The dimensions of CT specimen (unit: mm); (**b**) The photograph of CT specimen.

According to ASTM E647-24 standards [23], compact tension (CT) specimens of 304 stainless steel (304SS) with a thickness of 5 mm were processed as the substrate for laser repair of cracks. The dimensions and photograph of the specimen are shown in Figure 1. For a detailed description of the chemical composition of the specimen, refer to Reference [24]. A notch measuring 2 mm in length and 0.2 mm in width was created to simulate a crack using a wire electric discharge machining system. During the laser repair process, the entire 2 mm notch was effectively repaired.

The micro/nano alloy powder utilized in the laser repair experiment is composed of 3 wt.% nano WC, 1 wt.% nano Al_2_O_3_, 0.5 wt.% V and 304 stainless steel powder. The particle sizes of 304SS powder, WC, Al_2_O_3_ and V powder are within the ranges of 30–50 μm, 40–50 nm, 30–50 nm, and 20–25 μm, respectively. All powder materials are provided by Shanghai Xiangtian Nano Materials Co., Ltd., Shanghai, China.

The laser repair experiment was carried out by a CO_2_ laser. The mode of the laser is multi-mode, and the light intensity is approximately circular and evenly distributed. The light intensity outside the spot is null, and the heat flux density of circular laser spot is:

(1) $$q(r)=\frac{P}{\pi R^{2}}$$

where *q* is the heat flux of internal heat source, *r* is the distance from the spot center; *P* represents the laser power; and *R* is the spot diameter.

With a laser spot diameter D of 3 mm, a laser crack repair experiment with different laser power *P* and laser heating time t are arranged as follows:

(1) Select laser heating time *t* as 1.0 s and laser power *P* as 1600 W, 1800 W, and 2000 W, respectively.
(2) Select laser power *P* as 1800 W and laser heating time *t* as 0.5 s, 1.0 s, and 2.0 s, respectively.

**Figure 2:**
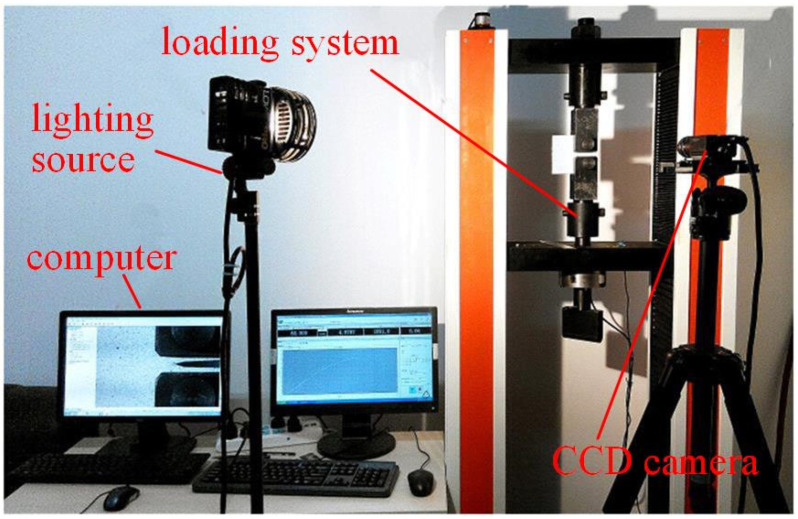
CT test device.

After repairing, the repair effect was studied by tensile test with the repaired CT specimen. Real time variation of displacement field of specimen with the load was recorded by a digital image correlation (DIC) system during the tensile test. Figure 2 shows the experimental setup.

After cutting, grinding, polishing and corrosion, metallographic specimens were prepared. The microstructure of the specimen was analyzed using a scanning electron microscope (SEM) Q45, which was produced by FEI Company, Hillsboro, OR, USA.

### 2.2. Trans-Scale Calculation Model

**Figure 3:**
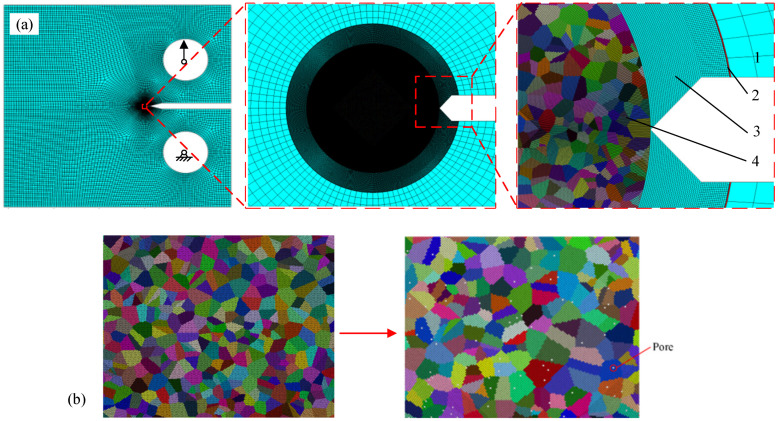
Trans-scale calculation model for CT specimen with pores. (**a**) Overall model, (**b**) schematic diagram illustrating the process of incorporating pores into the model.

Figure 3a illustrates the macro–micro trans-scale model of the laser repaired specimen. The model can be divided into four parts. Part “1” is established using conventional finite elements with an average element size of 0.5 mm, representing the peripheral part of the CT specimen used for applying loads and boundary conditions. The element type is a four-node plane stress element. Part “2” is constructed using Voronoi tessellations and crystal plasticity finite elements with an average element size of 0.98 μm, simulating the stress concentration region at the crack tip. Part “3” represents the variable-node elements used to connect the fine-scale elements and macro-scale elements. Part “4” is composed of four-node plane stress elements with an average size of 1 μm. The addition of these elements serves to enclose the model corresponding to “2” into a regular shape, thereby facilitating the establishment of multi-scale models. Referring to the method proposed by Aghababaei and Joshi [25], the model with pores in the repaired layer is established by removing several elements. Figure 3b indicates a partially enlarged model at the repaired layer, where white holes are used to represent the randomly distributed pores.

To conform to the loading condition in the tensile test, one loading hole is fixed, while the other loading hole is subjected to tensile load. The variable-node elements, i.e., the element indicated by “3”, is realized by a 13-node element through the UELMAT subroutine in ABAQUS (v. 2017) [21]. The material model of the repaired zone is realized by crystal plasticity theory through UMAT subroutine in ABAQUS [21]. The orientation of the crystals is assumed to be random. The materials of other regions are exposed by piecewise linear/power hardening materials [22]. The crystal slip hardening modulus adopts the simple form proposed by Peirce et al. [26] and is expressed as:

(2) $$h_{\alpha \alpha }=h\left(\gamma \right)=h_{0}{\mathrm{sech}}^{2}\left|\frac{h_{0}\gamma }{\tau_{s}-\tau_{0}}\right|$$

(3) $$h_{\alpha \beta }=qh\left(\gamma \right)(\alpha \neq \beta )$$

where $h_{\alpha \beta }$ is the slip hardening modulus, which is divided into self-hardening modulus $h_{\alpha \alpha }$ and latent hardening modulus $h_{\alpha \beta }(\alpha \neq \beta )$; γ is the cumulative shear strain; $h_{0}$ is the initial hardening modulus; $\tau_{s}$ is the saturation stress; and $\tau_{0}$ is the initial yield stress. For simplicity, the constant *q* is set to unity [27]. The initial hardening modulus $h_{0}$, the saturation stress $\tau_{s}$ and the initial yield stress $\tau_{0}$ of 304 stainless steel substrate with grain of 25 μm are obtained through inversion of the tensile stress–strain curve [21], as shown in Table 1.

## 3. Results

### 3.1. Experimental Results

**Figure 4:**
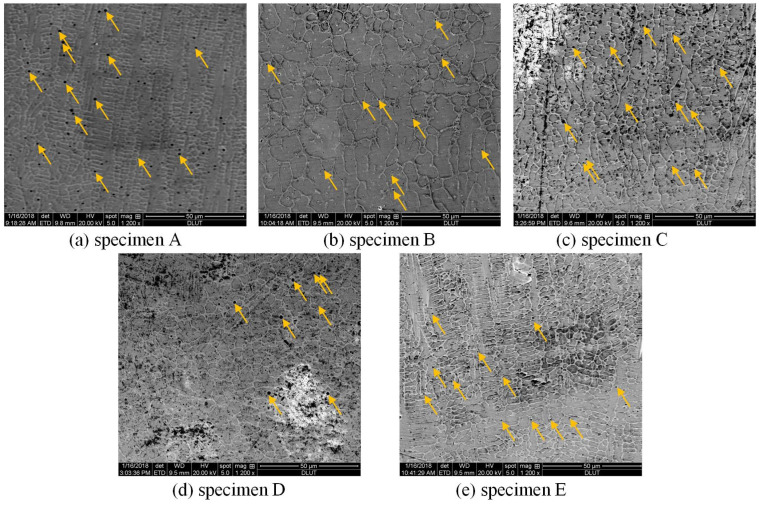
Microstructures at the repaired layer, with pores in the microstructure indicated by yellow arrows [20].

Figure 4 shows the microstructure of five specimens repaired by different laser power and heating time, with the pores in the microstructure indicated by arrows [20]. The laser process parameters used for these specimens and corresponding microstructure characteristics are listed in Table 2. When laser power is small, e.g., 1600 W, the grains are relatively fine, but a large number of pores appeared in the repaired layer, as shown in Figure 4a. When laser power increases to 1800 W, the dendrite began to transform to equiaxed grains, the overall microstructure is compact, and the number of pores in the repaired layer decreased (see Figure 4b). When laser power is too high, e.g., 2000 W, columnar dendrites appear in the repaired layer, the grain size and the number of pores increases (see Figure 4c). The effect of heating time on the microstructure is visible in Figure 4b,d,e. When the laser heating time is 0.5 s, as shown in Figure 4d, the grains in the repaired layer are small and the number of pores is low. When the laser heating time increases to 1.0 s, the grains in the repaired layer grow larger and the number of pores increases (see Figure 4b). When the laser heating time further increases to 2.0 s, the grain size and the number of pores in the repaired layer continue to increase (see Figure 4e). The grain size and porosity of the repaired layer were calculated by ImageJ software (v. 1.51j8) [28], as listed in Table 2.

### 3.2. Numerical Study on the Influence of Grain Size on Repair Quality

Phan et al. [29] applied crystal plasticity theory to study the effect of microstructure on mechanical behavior, and concluded that grain size has no effect on initial hardening modulus $h_{0}$ and the saturation stress $\tau_{s}$ but only on critical resolved shear stress $\tau_{0}$.

Weng et al. [30] provided the relationship between critical resolved shear stress $\tau_{0}$ and grain size *a*:

(4) $$\tau_{0}=\tau_{0}^{\infty }+k_{0}a^{-1/2}$$

where $\tau_{0}^{\infty }$ is the initial critical resolved stress in a free single crystal and $k_{0}$ is the Hall–Petch constant.

From macro scale, the Hall–Petch relationship can represent the relationship between material strength $\sigma_{\mathrm{HP}}$ and friction stress $\sigma_{0}$, Hall–Petch coefficient *k*, and grain size *a*:

(5) $$\sigma_{\mathrm{HP}}=\sigma_{0}+ka^{-1/2}$$

For 304 austenitic stainless steel, the friction stress $\sigma_{0}$ and the Hall–Petch coefficient *k* are 180 MPa and $7.589\;\mathrm{MPa}\cdot {\mathrm{mm}}^{0.5}$, respectively [31].

Thus, the critical resolved shear stress $\tau_{0}$ is inverted with the goal of minimizing the error between the simulated yield stress $\sigma_{m}$ and the calculated yield stress $\sigma_{\mathrm{HP}}$ by a CPFE model of tensile specimens. The computed results are presented in Table 3.

**Figure 5:**
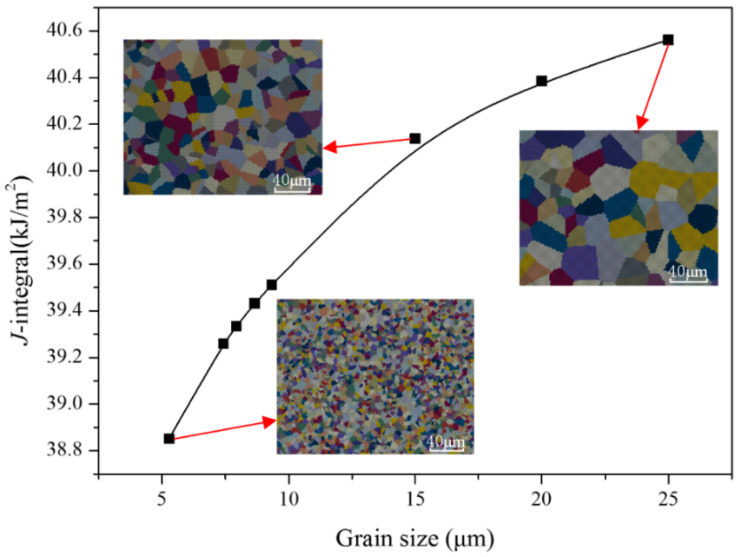
Variation of J-integral of the specimens with different grain sizes at repaired zone.

$\mathrm{Yield}\;\mathrm{stress}\;\sigma_{\mathrm{HP}}$ $\mathrm{Critical}\;\mathrm{resolved}\;\mathrm{shear}\;\mathrm{stress}\;\tau_{0}$ Figure 5 illustrates the J-integral of the specimens with varying grain sizes at repaired zone under a 20 kN load, calculated using the trans-scale calculation method. The grain distribution corresponding to some grain sizes is also shown in the figure. The figure clearly demonstrates that the J-integral increases as the grain size increases. Specifically considering the influence of grain size, specimens A, B, C, D, and E in Table 2 exhibit J-integral values that are 2.79%, 2.62%, 2.19%, 3.77%, and 2.38% lower, respectively, than those of specimens with a grain size of 25 μm (matching the grain size of 304SS substrate). During the laser repair process, the high energy density and rapid solidification rate prevent the grains in the repaired layer from growing sufficiently, leading to smaller grains. The refinement of grains results in an increased grain boundary area per unit volume. Grain boundaries serve as barriers to dislocation movement from one grain to another, leading to initial hardening. Moreover, smaller grain sizes correspond to a greater number of grain boundaries per unit volume and a reduced number of dislocations per unit length that need to be accommodated. Thus, the fewer opportunities there are for microcracks to nucleate and grow, the lower the internal stress of the material. Therefore, fine-grained structure is beneficial to the improvement of crack initiation resistance and the fracture properties of repaired parts.

### 3.3. Formatting of Mathematical Components

The individual CPS is an important parameter for describing crack propagation at the mesoscale. When the CPS on a slip system reaches a critical value, the crack will propagate along the slip system with the largest CPS [32]. The slip system with the largest CPS is called the dominant slip system. The individual CPS is characterized by its definition, which is:

(6) $$\gamma^{\left(\alpha \right)}=\int_{0}^{t}\left|{\dot{\gamma }}^{\left(\alpha \right)}\right|dt$$

where ${\dot{\gamma }}^{\left(\alpha \right)}$ is the slip rate on α slip system, t represents time.

**Figure 6:**
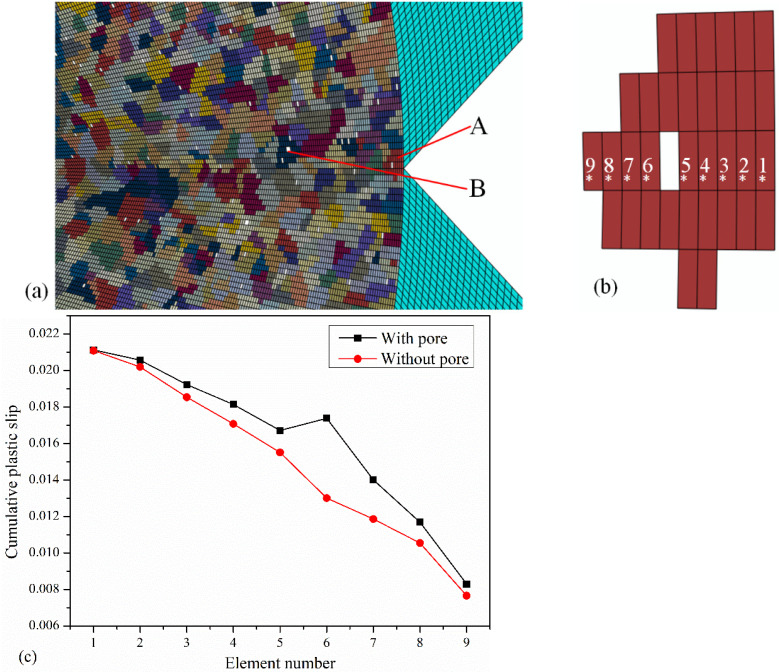
(**a**) The distribution of pores near the crack tip. (**b**) Grain A and element number. (**c**) CPS on dominant slip system near the pore along the front direction of the crack.

Figure 6 shows the distribution of pores near the crack tip of Specimen B simulated using a crystal plasticity model. The grain size and pore volume fraction of the repaired layer in the model are 7.93 μm and 0.5%, respectively, and the element size is 0.98 μm. Figure 6a shows the grains and pores near the crack tip; Figure 6b shows the positions of pores and elements on Grain A at the crack tip. Elements 1–9 are located along the direction of the crack front, and the position of their first integral point is indicated by a white “*” in the figure. The orientation of Grain A is (75°, 15°, 15°). The CPS on the dominant slip system with and without pores in Grain A is shown in Figure 6c. It is found that the CPS on dominant slip system with pores is greater than that without pores, and the closer to the position of pores, the greater the difference between them. The results show that the material more easily reaches the crack growth criterion under external load when pores are present—that is, the fracture properties decrease.

**Figure 7:**
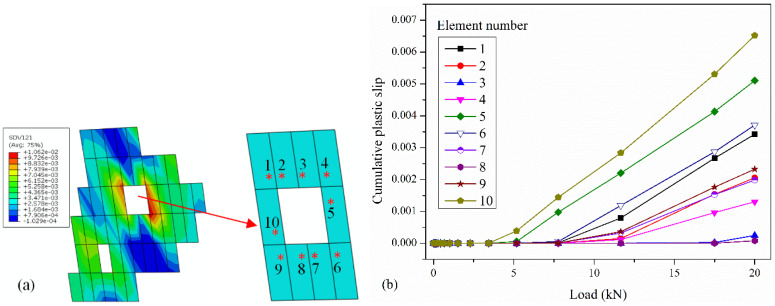
(**a**) Contour of CPS on all slip systems of grain B. (**b**) CPS on dominant slip system of each element near the large pore.

Figure 7a shows the CPS on all slip systems of grain B shown in Figure 6, Figure 7b shows the CPS on the dominant slip system of each element. The CPS data is extracted from the integration points marked with red “*” in Figure 7a. It is obvious that when the pore has sharp corners, the CPS is further increased, as shown in elements 1, 5, 6, and 10 in Figure 7b. Additionally, when the edges of pore are flat, the CPS is relatively small, as shown in elements 3 and 8 in Figure 7. Therefore, large pores or pores with sharp corners should be avoided in the repaired layer.

**Figure 8:**
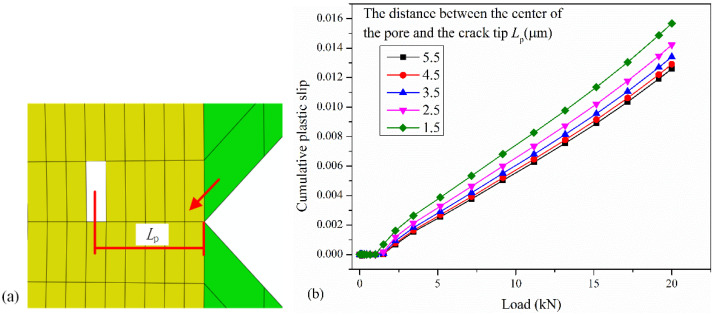
(**a**) Schematic of pore position. (**b**) The CPS on the dominant slip system varies with the position of pore.

Figure 8 shows the CPS on the dominant slip system near the crack tip when the position of the pore changes. The CPS on the dominant slip system at the integration point indicated by the red arrow in Figure 8a is used to investigate the changes in CPS. It can be seen from Figure 8b that the CPS near the crack tip gradually increases with decreasing distance from the porosity to the crack tip, and the magnitude of the increase is also increasing. In other words, the closer the pores in the repaired layer are to the crack tip, the better the crack repair quality.

### 3.4. Macro-Scale Analysis

**Figure 9:**
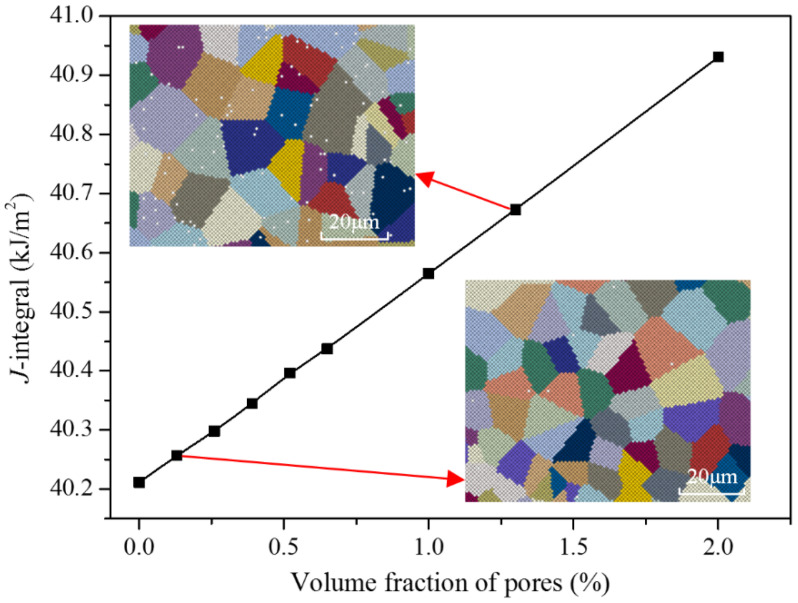
Variation of J-integral with volume fraction of pores under a 20 kN load.

Figure 9 illustrates the variation of J-integral with volume fractions of pores distributed in the repair layer under a 20 kN load. The grain distribution corresponding to some grain sizes is also shown in the figure. The calculations assume a pore radius of 0.98 μm. From the figure, it can be observed that within the calculated range, the J-integral linearly increases with the increase in the volume fraction of pores. When the volume fraction of pores in the repaired layer is 0.26% and 1.3%, the J-integral of the specimens under 20 kN load is increased by 0.2% and 1.1%, respectively, compared with those without pores. The linear relationship between J-integral and volume fraction of pores in the range of study indicates that the fracture properties of repaired parts will be significantly reduced in the presence of pores.

**Figure 10:**
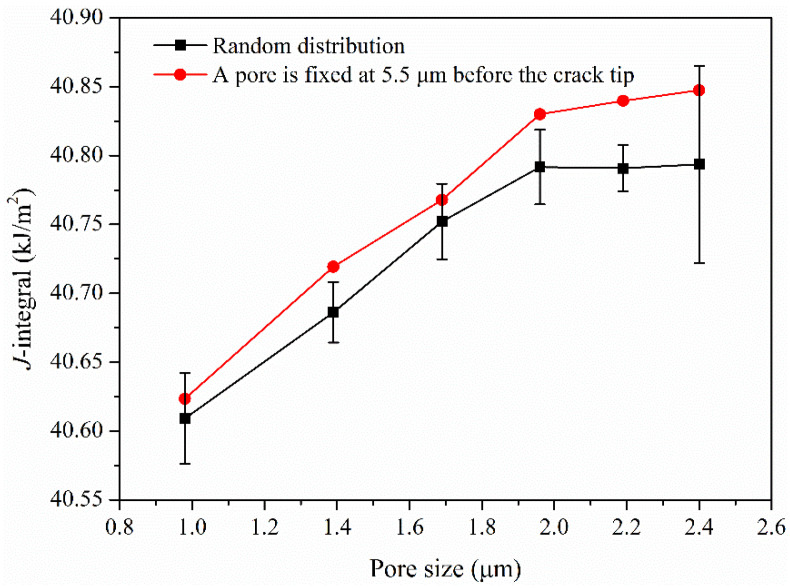
Variation of J-integral with pore size under a 20 kN load.

Figure 10 shows the variation of J-integral with the pore size at the load of 20 kN with the assumption that the volume fraction of pores is 1%. When the pore size is large, the J-integral will be affected by the position of pores. The influence of pore size on J-integral is studied by six calculations when the pores are randomly distributed. It can be observed that J-integral increases with the increase of pore size. When pore size reaches 2.4 μm, J-integral is greatly affected by pore position. On the basis of the random distribution of pores, one of pores is fixed at 5.5 μm before the crack tip, and the calculated J-integral is significantly greater than the average value of random distribution. Moreover, the larger the pore size, the greater the effect on J-integral. Therefore, when there are large pores in the repaired layer, especially near the crack tip, the fracture properties of repaired parts will be reduced significantly.

## 4. Discussion of Calculation and Experimental Results

**Figure 11:**
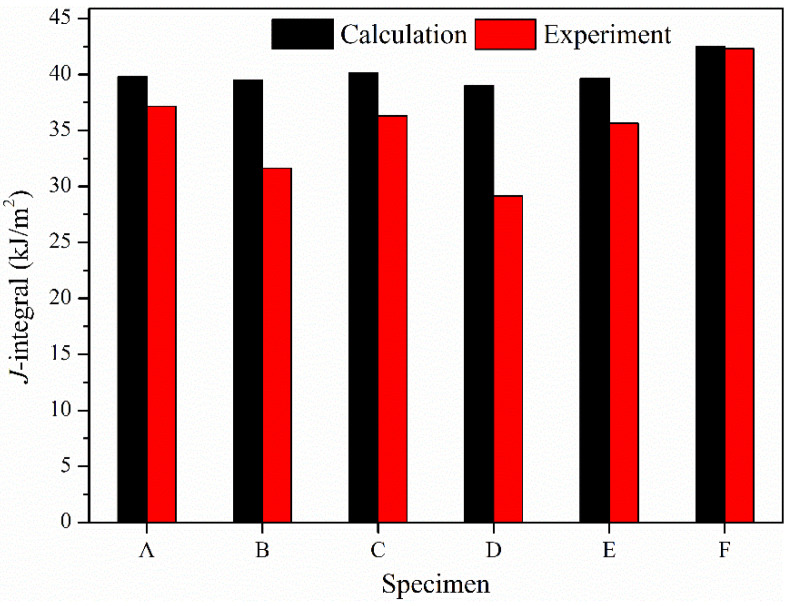
J-integral of repaired specimens obtained from calculation and experiment under a load of 20 kN.

Figure 11 shows the J-integral calculated using trans-scale model and the J-integral of different specimens under a load of 20 KN. The laser process parameters corresponding to Specimens A, B, C, D, and E are shown in Figure 4 and Table 2. Specimen F has not been repaired. The microstructure in the trans-scale model is also established based on the microstructure of Specimens A, B, C, D, E, and F. It can be seen that the laser process parameters of Specimen D are relatively suitable, the porosity is low, and the grain size is small. Correspondingly, the J-integral is more than 8% smaller than Specimen F and about 3% smaller than Specimen C. The laser power of Specimens A and C is either too small or too large, causing the porosity and grain size increase and the corresponding J-integral to increase. The heating time of specimens B and E is longer, the energy input for the molten pool is high, the porosity and grain size in the repaired layer increase, so the corresponding J-integral increases. Selecting appropriate laser process parameters can not only refine grain size but also reduce the volume fraction of pores in the repaired layer. Therefore, it is very important to select appropriate laser process parameters to improve repair quality.

## 5. Conclusions

In this article, meso–macro-mechanical analysis has been performed to study the influence of laser process parameters on the laser repair quality. The key findings can be summarized as follows:

When the laser power is 1800 W and the heating time is 0.5 s, the porosity of the repaired specimen is low, and the grain size is small. The corresponding J-integral is more than 8% smaller than that of the unrepaired specimen and approximately 3% smaller than that of the repaired specimen subjected to a laser power of 2000 W and a heating time of 1 s. Both excessive and insufficient laser power, as well as prolonged heating time, can result in increased porosity in the repair layer and a reduction in the fracture properties of the repaired components.

The pores in the repair layer enhanced the CPS of the surrounding crystals, particularly for pores with sharp corners, where the CPS exhibited a more pronounced increase. As the distance from the hole to the crack tip decreases, the CPS near the crack tip gradually rises, and the extent of this increase also becomes more significant.

The J-integral of the repaired specimen increases linearly with the pore volume fraction in the repair layer. By selecting appropriate laser processing parameters, the grain size can be refined and the pore volume fraction can be minimized, thereby reducing the J-integral and ultimately improving the repair quality.

The current established model primarily focuses on the influence of microstructural features, such as pores, on the fracture properties of repaired components. However, it has not fully addressed the complex mechanisms associated with the heat-affected zone (HAZ) during the laser repair process. As a critical area in this process, the microstructural and performance changes within the HAZ can significantly impact the overall properties of the repaired components. Therefore, including the HAZ into the research scope is essential for enhancing the properties evaluation and optimization of repair parts, and it is also a vital direction for future research. In addition, this study primarily focused on the microstructural characteristics of pores; however, it is important to note that other types of defects, such as microcracks, may also develop during laser repair processes. These defects can significantly affect the fracture properties of the repaired components. Due to limitations in experimental conditions and technical capabilities, it is currently not possible to fully capture and quantify the characteristics of these defects and their specific effects on material properties. Future research will concentrate on how laser processing parameters and material composition can mitigate the formation of microcracks in the repair layer and will as analyze the influence of microcracks on the fracture properties of the repaired components. By optimizing material composition and laser technology, the occurrence of microcracks in the repair layer can be reduced or eliminated, thereby enhancing the structural integrity and service life of the repaired parts.

## Figures and Tables

**Table 1:** Crystal plasticity constant of 304 stainless steel substrate.

$\mathrm{Initial}\;\mathrm{Hardening}\;\mathrm{Modulus}\;h_{0}$	$\mathrm{Saturation}\;\mathrm{Stress}\;\tau_{s}$	$\mathrm{Initial}\;\mathrm{Yield}\;\mathrm{Stress}\;\tau_{0}$
464.40 MPa	191.08 MPa	78.86 MPa

**Table 2:** Grain size and porosity of specimens repaired with different laser process parameters.

Specimen	A	B	C	D	E
P-D-T (W-mm-s)	1600-3-1	1800-3-1	2000-3-1	1800-3-0.5	1800-3-2
Grain size (μm)	7.42	7.93	9.33	5.27	8.65
Porosity (%)	1.50	0.50	1.76	0.44	0.63

**Table 3:** The calculated critical resolved shear stress $\tau_{0}$ of 304SS.

Grain Size (μm)	5	5.27	7.42	7.93	8.65	9.33	15	25
$\mathrm{Yield}\;\mathrm{stress}\;\sigma_{\mathrm{HP}}$ (MPa)	287.33	284.55	268.11	265.23	261.60	258.57	241.97	228.00
$\mathrm{Critical}\;\mathrm{resolved}\;\mathrm{shear}\;\mathrm{stress}\;\tau_{0}$ (MPa)	100.25	99.17	94.08	92.59	91.82	90.51	84.65	78.86

## Data Availability

Data are contained within the article.
